# A novel anatomical integrated acetabular plate for acetabular fracture involving posterior wall/column: a biomechanical study

**DOI:** 10.3389/fbioe.2025.1691895

**Published:** 2026-01-06

**Authors:** Xuan Pei, Yu Chen, Zhixun Fang, Ziren Xiong, Jianan Chen, Yifan Zheng, Ting Wang, Shenglong Qian, Long Chen, Guodong Wang, Jing Qi, Ximing Liu

**Affiliations:** 1 Department of Orthopeadics Surgery, General Hospital of Central Theater Command of PLA, Wuhan, China; 2 University Center of Orthopaedic, Trauma and Plastic Surgery, University Hospital Carl Gustav Carus at Technische Universität Dresden, Dresden, Germany; 3 Department of Traumatology, Hanchuan People’s Hospital, Hanchuan, China; 4 Department of Traditional Chinese Orthopedics and Traumatology, Xiamen Third Hospital, Xiamen, China; 5 Department of Orthopaedics, Union Hospital, Tongji Medical College, Huazhong University of Science and Technology, Wuhan, China; 6 Baiyun Branch, Nanfang Hospital, Southern Medical University, Guangzhou, China; 7 School of Medicine, Wuhan University of Science and Technology, Wuhan, China; 8 Medical School, University of Rostock, Rostock, Germany; 9 Hubei University of Chinese Medicine, Wuhan, China

**Keywords:** acetabular fractures, anatomically integrated acetabular plate, biomechanical, posterior column fracture, posterior wall fracture

## Abstract

**Introduction:**

The optimal treatment for complex acetabular fracture involving the posterior wall and column remains controversial. To address this issue, a novel anatomically integrated acetabular plate (AIP) was developed, designed to integrate the biomechanical advantages of both reconstruction and T-shaped plates. This biomechanical study aimed to evaluate the mechanical performance of the AIP in comparison with conventional fixation methods.

**Methods:**

Acetabular fractures involving the posterior wall and column were created in 18 fresh-frozen pelvis specimens and assigned to three fixation groups: (1) an anatomically integrated plate (AIP), (2) two reconstruction plates with a T-plate (RPTP), and (3) two reconstruction plates with two lag screws (RPLS). A standing position was simulated, and a Zwick Z100 testing machine applied an axial load from 0 to 1400 N. A load-displacement sensor and digital dial gauge were used to measure overall displacement, stiffness, and displacement of the posterior wall and column to evaluate the mechanical stability of each fixation construct.

**Results:**

Under increasing axial loading, all three groups of model specimens exhibited a linear trend in axial displacement without sudden load drops. Among the groups, the AIP group demonstrated the smallest overall displacement (1.87 ± 1.09 mm), followed by the RPTP (2.29 ± 1.12 mm) and RPLS groups (2.63 ± 1.21 mm). No significant difference in displacement was observed between the AIP and RPTP groups under loads of 0–1000 N (*P* > 0.05), whereas a significant difference emerged at higher loads of 1200–1400 N (*P* < 0.05). Under a peak load of 1400 N, the axial stiffness followed the trend: Normal (NOR) group > AIP group > RPTP group > RPLS group, with mean stiffness values of 356.10 ± 12.33 N/mm, 339.87 ± 21.86 N/mm, 302.04 ± 13.69 N/mm, and 266.32 ± 9.16 N/mm, respectively. The AIP group exhibited significantly higher stiffness than both the RPTP and RPLS groups (*P* < 0.05), with no significant difference between the AIP and NOR groups (*P* > 0.05). Furthermore, the AIP group showed significantly lower displacement of the acetabular posterior wall and column compared to the RPTP and RPLS groups (*P* < 0.05). Notably, two specimens in the RPLS group showed posterior wall displacements exceeding 2 mm, which met the criteria for internal fixation failure.

**Conclusion:**

Overall, the AIP group provided the best biomechanical performance in terms of minimizing displacement and maximizing stiffness, followed by RPTP and RPLS group, indicating its potential superiority for the stabilization of acetabular fractures involving the posterior wall and column.

## 1 Introduction

In the past 30 years, posterior acetabular fractures have become 2 to 3 times more common, likely due to the growing elderly population and their increasing engagement in physical activities (Krappinger et al., 2024; Butterwick et al., 2015). This rise in incidence is reflected in radiographic findings, which show that posterior wall fractures account for 20.9%–21% of cases, posterior column fractures for 1.3%–2%, and posterior column combined with posterior wall fractures (PCPWF) for 4.9%–6% (Kelly et al., 2020; Albrektsson et al., 2022). PCPWF typically result from a posteriorly directed force applied to the femoral head, usually when the hip is in a flexed and adducted position; The force initially causes a posterior wall fracture and, if sufficient, may propagate to involve the posterior column (Zhang et al., 2025; Zhang et al., 2024). Due to the complexity and instability of these fractures, surgical intervention is necessary to achieve anatomical reduction and stable fixation, which are essential for restoring the biomechanical function of the hip joint and minimizing the risk of long-term complications (Zhang et al., 2025; Audretsch et al., 2022; Matta, 1996; Chen et al., 2020).

Traditionally, the combination of reconstruction plates and interfragmentary screws was the most commonly used method for treating PCPWF (Yu et al., 2004). Later, to improve the stability of the posterior wall of the acetabulum, reconstruction plates in combination with spring plates or T-shaped plates were popularized for PCPWF (De Mauro et al., 2023; Cho et al., 2019). However, this approach also presents certain challenges, primarily related to biomechanical instability resulting from repeated bending of the plates and improper placement of the hardware. More recently, biomechanical investigations have focused on improving fixation strength and reducing implant-related complications through anatomical plate design and precontoured reconstruction systems. For instance, Zhang et al. designed an H-shaped anatomical titanium plate (HTP) and demonstrated superior biomechanical stability compared with conventional reconstruction plate constructs, as well as favorable clinical outcomes with a high rate of anatomic reduction and low complication incidence (Zhang et al., 2025). In addition, Hakim et al. performed a finite element analysis of a newly designed acetabular plate that combined the functions of a spring plate and a reconstruction plate. Their results showed that the construct effectively restored stress distribution and deformation patterns in fractured pelvises to those of the normal pelvis, indicating promising biomechanical performance (Hakim et al., 2025). Building on these developments, we designed a novel anatomically integrated acetabular plate (AIP) that integrates the functional advantages of reconstruction and T-shaped plates, aiming to overcome the limitations of conventional fixation techniques and to enhance mechanical stability and ease of fixation. This design has been granted a national invention patent (Patent No. ZL 2023 2 2817309.7).

The present study aimed to compare the mechanical stability of the newly developed AIP with that of reconstruction plates combined with T-shaped or two lag screws for the treatment of PCPWF. We hypothesized that the AIP would provide superior or at least comparable mechanical stability to conventional fixation methods, particularly in terms of overall stiffness and displacement of the reconstructed pelvic models. Therefore, the present study adds to the existing biomechanical evidence on acetabular fracture fixation and supports the optimization of fixation strategies for this fracture type.

## 2 Materials and methods

This study complied with the protocols approved by the Ethics Committee of the General Hospital of Central Theater Command of PLA (No. [2022]-027-01).

### 2.1 Preparation and preservation of specimens

Eighteen fresh-frozen pelvic specimens were provided by the Department of Anatomy, Wuhan University. Each specimen extended from the fourth lumbar vertebra to the proximal third of both femurs, with the periosteum and hip joint capsules intact. An *a priori* power analysis (G*Power 3.1; Heinrich Heine University, Düsseldorf, Germany) for a one-way ANOVA (three groups) assuming *f* = 0.75, α = 0.05, and power = 0.80 indicated that 21 specimens (7 per group) were required. However, due to the limited availability of and the ethical constraints associated with fresh-frozen human cadaveric specimens, 18 specimens (6 per group) were used, providing an actual power of 0.79. This sample size is consistent with previous cadaveric biomechanical studies of acetabular fixation, which typically include 4–6 specimens per group (Wu, 2019; Chung et al., 2024). Radiographic examinations were performed to exclude any abnormalities such as fractures, deformities, rheumatic diseases, tuberculosis, neoplasms, or degenerative changes. Quantitative computed tomography (QCT) measurements confirmed that all donor specimens had normal bone density without signs of osteoporosis, and there were no significant differences in bone mineral density (BMD) among the three groups (p > 0.05), indicating comparable bone quality across specimens. After thawing, the acetabulum, femoral shafts, and the soft tissues and muscles attached with the lumbar vertebrae were carefully removed by dissection. At the same time, the ligaments connecting the fifth lumbar vertebra to the pelvis and sacrum, as well as the bilateral hip joint capsules and the pubic symphysis, were fully preserved. The samples were stored at −20 °C and thawed at room temperature 24 h before biomechanical testing. Throughout both the pre-experimental and experimental phases, the tissues were kept moist with saline-soaked gauze to prevent dehydration.

### 2.2 Development of PCPWF model and three types of fixation strategies

**FIGURE 1:**
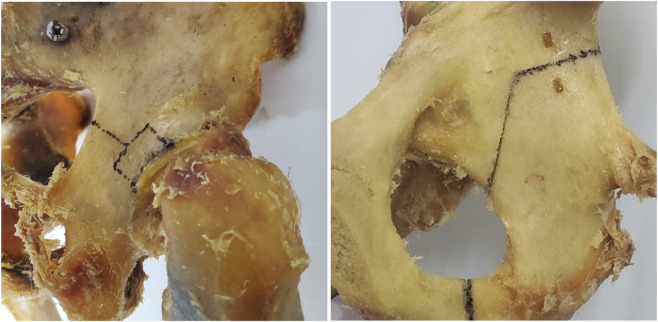
Typical acetabular posterior column and posterior wall fracture model.

**FIGURE 2:**
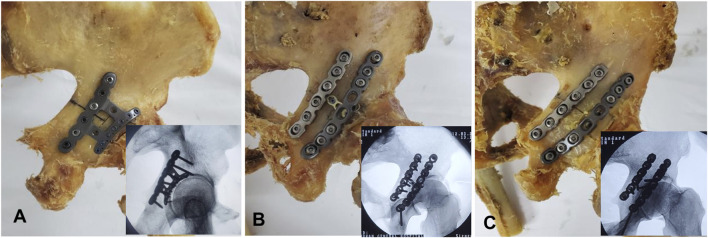
Photographs and radiographic imaging showing the three configurations used for APCWF fixation. **(A)** an anatomically integrated plate; **(B)** 6-hole and 8-hole 3.5 mm conventional reconstruction plates combined with a T-plate; **(C)** 6-hole and 8-hole 3.5 mm conventional reconstruction plates combined with two lag screws.

Referring to the studies by Altun (Altun et al., 2019) and Şibar (Şibar et al., 2023), a model of a typical acetabular fracture (acetabular fracture involving posterior wall/column) (Figure 1) was created by electric drills, kirschner wires, and oscillating saws. All samples were randomly divided into 3 groups (n = 6 per group). According to the principles of fracture reduction for this fracture type, the posterior column fracture is first reduced to restore the overall integrity of the acetabular posterior structure. Subsequently, the femoral head is used as a template to reduce the posterior wall fracture fragments. Each group received a different internal fixation strategy to stabilize the fracture model. AIP group: This group employed a specially designed plate system in which the main plate for the posterior column and the subplate for the posterior wall were connected via a transverse linking plate, achieving “posterior column-posterior wall integrated fixation” (Figure 2A). Two reconstruction plates with a T-plate (RPTP) group: This group employed 3.5-mm conventional reconstruction plates (one 6-hole and one 8-hole) combined with a T-plate (Figure 2B). Two reconstruction plates with two lag screws (RPLS) group: This group employed 3.5-mm conventional reconstruction plates (one 6-hole and one 8-hole) combined with two lag screws (outside the plate) (Figure 2C). The accuracy of implant placement and the effectiveness of fracture fixation were verified using radiographic imaging.

### 2.3 Biomechanical tests

**FIGURE 3:**
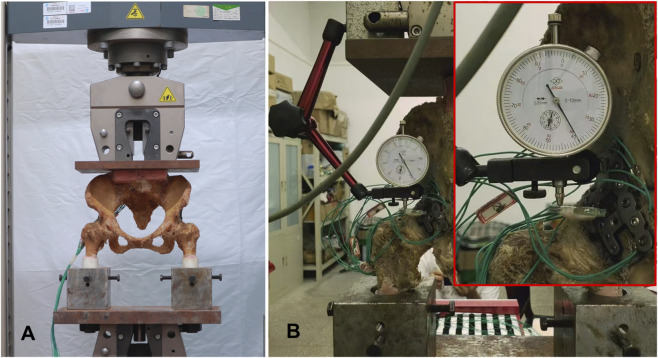
Experimental setup. **(A)** Specimen positioned to simulate standing posture for biomechanical testing. **(B)** Multifunctional digital micrometer used to record displacement of the acetabular posterior wall and posterior column during loading.

Mechanical loading was conducted using a Zwick Z100 testing machine (Zwick, Germany) (Figure 3A), following a loading protocol similar to that described by Su et al. (Su et al., 2017) and Zhang et al. (Zhang et al., 2013). A plane defined by the bilateral anterior superior iliac spines and the pubic symphysis was oriented perpendicular to the ground. Each femur was positioned at 15° of internal rotation and 20° of abduction relative to the pelvic sagittal plane to simulate a physiological standing posture. The angles were adjusted using a digital goniometer (accuracy ±0.5°) and maintained with a custom-made alignment jig during potting. The distal femur was embedded in epoxy resin while maintaining the alignment, and the final orientation was verified within ±2° using a digital goniometer. The preprocessing loads ranging from 0 to 300 N were applied at a constant displacement rate of 5 mm/min. The axial load was gradually increased from 0 N to 1400 N at a constant displacement rate of 5 mm/min to simulate physiological loading conditions. For each loading test, load and displacement data were recorded through a computer connected to the displacement sensor of the Zwick Z100 testing machine, and the corresponding load-displacement curves were subsequently generated. According to the method described by Wu et al., the displacements of posterior wall and posterior column of the acetabulum during loading were measured using a multifunctional digital dial gauge (Beijing Deli love Monitoring Technology, Beijing, China) (Figure 3B) (Wu et al., 2021). The biomechanical experiments were conducted in the Biomechanics Laboratory at Wuhan University of Technology.

### 2.4 Data collection

During the standing axial compression test (0–1400 N), the overall pelvic axial displacement and the displacements of the posterior wall and posterior column were measured for each specimen. The load-displacement curve was plotted, and the slope of the curve represents the overall axial stiffness of the pelvis (N/mm). The quality of fracture reduction and fixation failure were evaluated according to the criteria established by Matta and Bray (Matta, 1996; Bray et al., 1987). Implant failure was defined as a displacement greater than 2 mm in the posterior wall or posterior column (Chung et al., 2024).

### 2.5 Statistical analysis

Data are presented as the mean ± standard deviation. Displacement data among different experimental groups were analyzed using one-way analysis of variance (ANOVA) at a 95% confidence level. When variances were homogeneous, Tukey’s test was applied; when variances were unequal, the Kruskal–Wallis test was used. All analyses were performed using GraphPad Prism (version 10.1.2; GraphPad Software, San Diego, CA, USA), with a significance level set at *P* < 0.05.

## 3 Results

### 3.1 Analyze the overall displacement of the structure

**FIGURE 4:**
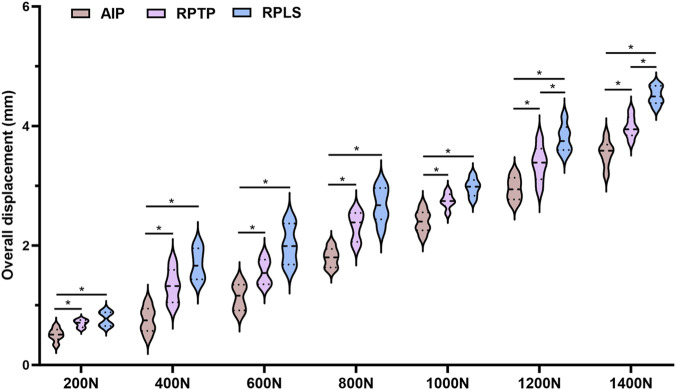
Violin plot showing overall displacement under 0–1400 N load (*p < 0.05).

As the axial load increased from 0 N to 1400 N, the axial compression displacements of the three groups of model specimens exhibited a linear increasing trend, as shown in Figure 4. None of the groups showed a sudden load drop during loading. The overall displacement followed the order: AIP group (1.87 ± 1.09 mm) < RPTP group (2.29 ± 1.12 mm) < RPLS group (2.63 ± 1.21 mm). The overall axial displacement in the RPLS group was significantly greater than that in the AIP group and the RPTP group, and the differences were statistically significant (*P* < 0.05). There was no significant difference in the overall displacement of the model between the AIP and RPTP group under a load of 0–1000 N (*P* > 0.05). However, at 1200–1400 N, the difference was statistically significant (*P* < 0.05).

### 3.2 Analyze the overall stiffness of the structure

**FIGURE 5:**
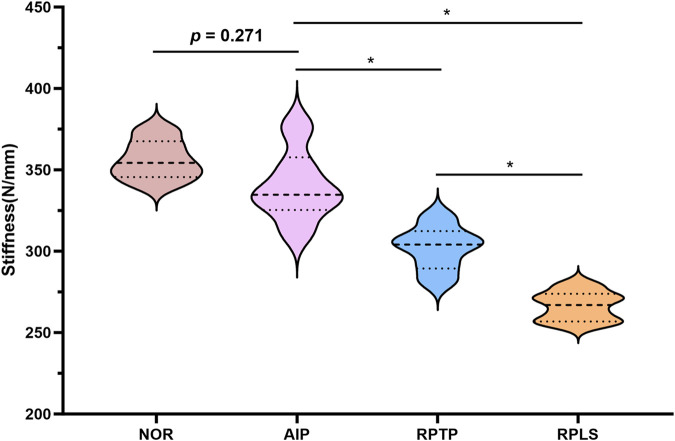
Average axial stiffness of fixation constructs under a 1400 N compressive force (*p < 0.05).

Under an axial load of 1400 N, the axial stiffness of the model in each group showed a consistent trend: Normal (NOR) group > AIP group > RPTP group > RPLS group (Figure 5). The mean stiffness values of the NOR group, AIP group, the RPTP group, and the RPLS group were 356.10 ± 12.33 N/mm, 339.87 ± 21.86 N/mm, 302.04 ± 13.69 N/mm, and 266.32 ± 9.16 N/mm, respectively. The axial stiffness of the AIP group was significantly higher than that both of the RPTP group and RPLS groups (*P* < 0.05). No significant difference in overall stiffness was observed between the NOR and AIP groups under the 1400 N load (*P* > 0.05). In addition, the RPTP group exhibited significantly greater stiffness than the RPLS group (*P* < 0.05).

### 3.3 Measurement of displacement in acetabular posterior wall and column

**FIGURE 6:**
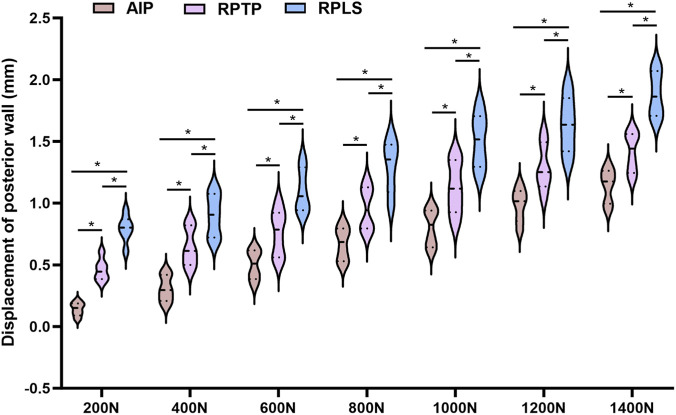
Violin plot showing average displacement of posterior wall under 0–1400 N load (*p < 0.05).

**FIGURE 7:**
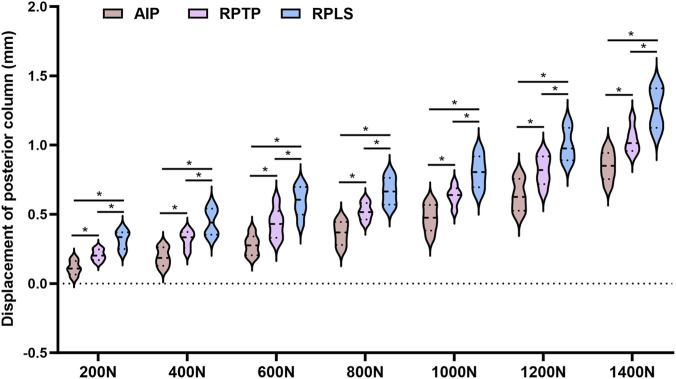
Violin plot showing average displacement of posterior column under 0–1400 N load test (*p < 0.05).

Model specimens in each group were subjected to axial mechanical loading ranging from 0 to 1400 N in the standing position, and the displacements of the posterior wall fracture fragment and the posterior column were measured using a multifunctional digital dial gauge. The results are presented in Figures 6, 7. The mean displacement values of the posterior wall and posterior column fracture fragments among the three groups were ranked in ascending order as follows: AIP group < RPTP group < RPLS group. After loading was applied, the average displacement values of the posterior wall and posterior column in the AIP group were significantly smaller than those in the RPTP and RPLS groups (*P* < 0.05). In the RPLS group, 2 model specimens exhibited posterior wall displacement exceeding 2 mm, which met the criteria for internal fixation failure.

## 4 Discussion

Acetabular fractures remain a major challenge in orthopedic trauma care, with a steadily increasing incidence attributed to the rising prevalence of high-energy trauma in recent years (Butterwick et al., 2015; Antell et al., 2017). Surgical intervention aims not only to achieve anatomic reduction of the hip joint but also to provide stable internal fixation, which is essential for maintaining reduction and enabling early, pain-free mobilization during fracture healing (May et al., 2018; Tannast et al., 2012). For posterior wall and posterior column fractures, reconstruction plates, lag screws, spring plates, and any combination of these are the main treatment options (Yu et al., 2004; De Mauro et al., 2023; Cho et al., 2019). Although widely adopted, these techniques for acetabular fractures fixation have several limitations. For example, reconstruction plates combined with lag screws carry an increased risk of intra-articular penetration, show poor adaptability for comminuted fracture patterns, and present technical difficulties due to anatomical variability and poor plate conformity (Hakim et al., 2025; Ebraheim et al., 1989). Therefore, an anatomically integrated acetabular plate was designed for the treatment of APCWF to address these limitations. However, to date, biomechanical evidence supporting its mechanical stability remains lacking. This study was conducted to evaluate whether the newly designed AIP provides mechanical stability comparable to that of conventional fixation techniques.

Based on the loading protocols proposed by Su et al. and Zhang et al., we applied a peak load of 1400 N, corresponding to approximately twice the average body weight, to simulate a double-limb standing posture (Zhang et al., 2013; Albrektsson et al., 2023). This load represents a physiologically high loading condition that challenges the stability of the acetabular fixation, and was therefore defined as the worst-case condition in the present biomechanical test. Based on this approach, static axial loads ranging from 0 to 1400 N were gradually applied to the pelvic specimens. The load-displacement curves were recorded, reflecting the relationship between applied load and overall displacement. The results showed that the overall displacement increased linearly with the increase in axial loading force. In this study, during the early loading phase (0–1000 N), there was no significant difference in overall displacement between the AIP and RPTP groups, indicating comparable stability. However, as the loading force increased (1200–1400 N), the AIP group exhibited a smaller overall displacement than the RPTP group. These findings suggest that during early-stage partial weight-bearing, there is no significant difference in the stability between the two fixation systems. However, under full weight-bearing conditions (1400), the AIP group demonstrated greater stability, likely due to its integrated design.

Previous studies have shown that accurate fracture reduction and stable fixation of fracture fragments are key factors in minimizing the incidence of post-traumatic arthritis and cartilage degeneration in the hip joint (Jude et al., 1964; Graul et al., 2022). In the present study, the AIP group exhibited significantly less displacement of the posterior wall and posterior column compared to the RPTP and RPLS groups. These results suggest that the AIP group effectively maintains fracture reduction, thereby contributing to the restoration of normal hip joint biomechanics and improving overall joint stability. A possible explanation is that the AIP, featuring multiple locking screws in the subplate, provides multi-point anchorage of posterior wall fragments, thereby reducing fracture displacement and enhancing fixation stability. Traditional internal fixation constructs often fail to provide sufficient stability for complex or comminuted fractures. In contrast, the AIP incorporates locking holes that provide angular stability, and the risk of intra-articular screw penetration can be minimized by avoiding screw placement within the acetabular danger zone. Furthermore, owing to its superior conformity to the anatomical features of the posterior column and posterior wall of the acetabulum, the AIP enables more accurate fracture reduction. Liu and Graul et al. also reported that internal fixation systems with better conformity to the anatomical morphology of the acetabulum exhibit greater biomechanical stability, effectively reduce stress concentration, and decrease the risk of implant failure (Liu et al., 2023; Liu et al., 2015). Furthermore, the displacements of the posterior wall and posterior column in the RPLS group were greater than that in the RPTP group (*P* < 0.05). In our previous study, we used the finite element analysis to evaluate the biomechanical properties of the two fixation systems (Hinz et al., 2022), and the results were consistent with the current study.

Stiffness reflects the ability of a fixation construct to resist deformation under load and serves as a key indicator of mechanical stability in biomechanical studies ^32^. In this study, the AIP group represented to an anatomically integrated acetabular plate that connects the posterior column main plate to the posterior wall subplate via a transverse linking component, thereby achieving a combined fixation of the posterior column and posterior wall. This innovative design facilitates a more uniform load distribution across the internal fixation system, reduces stress concentration, and significantly enhances the stability of fracture fragments. Under a load of 1400 N, the AIP fixation system demonstrated greater stiffness compared to the RPTP and RPLS groups. The RPLS group exhibited the lowest axial stiffness, indicating limited resistance to deformation and a higher the risk of fixation failure, particularly under high-load conditions. Two specimens in the RPLS group exhibited posterior wall displacement exceeding 2 mm, which was indicative of failure of internal fixation. To exclude the potential influence of bone quality on these mechanical differences, QCT was performed. The measurements confirmed that all donor specimens had normal bone density without signs of osteoporosis, and no significant differences in BMD were found among the three groups (*p* > 0.05). These results indicate that the observed differences in stiffness and fixation stability were primarily attributable to the characteristics of the fixation constructs rather than variations in bone quality. These findings indicate that the differences in stiffness and fixation stability were mainly attributable to the characteristics and structural design of the fixation constructs, supporting the reliability of the comparative analysis.

This study has several limitations. First, biomechanical testing was conducted using six fresh-frozen specimens per group, which may have limited the statistical power of the results and potentially masked subtle differences between fixation methods. Larger sample sizes are needed to enhance the statistical power and reliability of the findings. Second, acetabular fractures were created using surgical instruments, resulting in minimal fragment displacement and muscles were removed to facilitate measurement. These simplifications may have led to a more stable construct than typically occurs *in vivo*, potentially underestimating the challenges of fixation in clinical scenarios. Third, fracture displacement occurs in three dimensions, but the dial gauge measured only predefined points, which may have overlooked complex spatial deformation. Future studies should use three-dimensional (3D) motion capture or digital image correlation (DIC) to obtain more complete spatial, and develop fracture models with controlled displacement, preserved soft tissues, while applying finite element analysis (FEA) to better represent soft-tissue effects and enhance the translational relevance of the findings.

## 5 Conclusion

Based on the results of this *in vitro* biomechanical study, we conclude that the AIP group provides a more effective fixation construct for PCPWF compared to the RPTP and RPLS groups. Therefore, the AIP group may serve as a viable alternative for the surgical treatment of PCPWF.

## Data Availability

The original contributions presented in the study are included in the article/supplementary material, further inquiries can be directed to the corresponding author.
